# RNA stability is regulated by both RNA polyadenylation and ATP levels, linking RNA and energy metabolisms in *Escherichia coli*

**DOI:** 10.1128/mbio.02680-24

**Published:** 2024-11-29

**Authors:** Charlotte Roux, Marvin Ramos-Hue, Marjorie Audonnet, Marie-Pierre Duviau, Sébastien Nouaille, Agamemnon J. Carpousis, Sandrine Laguerre, Eliane Hajnsdorf, Muriel Cocaign-Bousquet, Laurence Girbal

**Affiliations:** 1 TBI, Université de Toulouse, CNRS, INRAE, INSA, Toulouse, France; 2 UMR8261, CNRS, Université de Paris, Institut de Biologie Physico-Chimique, Paris, France; The Pennsylvania State University, University Park, Pennsylvania, USA

**Keywords:** RNA polyadenylation, PAP I, RNA stability, energy, ATP, *Escherichia coli*

## Abstract

The post-transcriptional process of RNA polyadenylation sits at the crossroads of energy metabolism and RNA metabolism. RNA polyadenylation is catalyzed by poly(A) polymerases, which use ATP as a substrate to add adenine to the 3′ end of RNAs, which can alter their stability. In *Escherichia coli,* RNA polyadenylation mediated by the major poly(A) polymerase was previously shown to facilitate degradation of individual RNAs. In this study, we performed the first genome-wide study of RNA stability in the absence of PAP I. Inactivation of the *pcnB* gene coding for PAP I led to the stabilization of more than a thousand of *E. coli* RNAs in the form of full-length functional molecules or non-functional fragments. The absence of PAP I altered the energy metabolism, with an almost 20% reduction in ATP levels. To better understand how RNA and energy metabolisms are interconnected, we investigated the role of ATP levels in regulating RNA stability. When we lowered intracellular ATP levels below 0.5 mM, many RNAs were stabilized, demonstrating the causal link between ATP levels and RNA stability for the first time in *E. coli*. Above this concentration, changes in ATP levels had no impact on RNA stability. We also demonstrated that some RNAs were stabilized when PAP I was inactivated by low ATP availability. These results clearly demonstrate that PAP I mediates an energy-dependent RNA stabilization, which may contribute to cell energy homeostasis under energy-limited conditions.

**IMPORTANCE:**

Poly(A) polymerases are prime targets for understanding the interactions between RNA polyadenylation, RNA stability, and cellular energy. These enzymes catalyze the process of RNA polyadenylation, which involves ATP hydrolysis and addition of poly(A) tails to the 3′ end of RNAs. 3′ end poly(A) extensions potentially facilitate RNA degradation in bacteria. In this study, we inactivated the *pcnB* gene encoding PAP I, the major poly(A) polymerase in *E. coli*, and investigated the effects on RNA stability and energy levels. Our results show for the first time in *E. coli* a genome-wide RNA stabilization in the absence of PAP I associated with a decrease in ATP levels. We provide the first evidence in *E. coli* of a link between ATP levels and RNA stabilization and demonstrate that this is mediated in some cases by PAP I. PAP I-mediated RNA stabilization at low ATP levels could be a means of energy conservation under energy-limited conditions.

## INTRODUCTION

RNA degradation is a ubiquitous process that, in most cases, begins in *Escherichia coli* with endoribonucleolytic cleavages mainly performed by RNase E, RNase III, RNase G, RNase P, RNase LS, or RNase Z. 3′ end exoribonucleases (polynucleotide phosphorylase [PNPase], RNase II, and RNase R) then convert the endonucleolytic cleavage products to oligonucleotides that are further degraded to mononucleotides by oligoribonuclease (see references 1–5 for reviews). In some cases, RNA degradation is initiated by 3′ end exoribonucleases (6, 7). RNA degradation can be facilitated by the addition of 3′ end poly(A) tails by poly(A) polymerase I (PAP I), which provides a binding site for 3′ exoribonucleases (8, 9). So far, the effect of PAP I-mediated polyadenylation on RNA stability has only been studied for individual RNAs. The function of the *pcnB* gene, which codes for PAP I, was first identified in studies of plasmid copy number regulation. Antisense RNA I of ColE1-type plasmids and *copA* sRNA of plasmid R1 are destabilized by PAP I-dependent polyadenylation (10–12). PAP I polyadenylation also affects tRNA maturation and degradation because it competes with 3′ exoribonucleases for access to the 3′ end termini of pre-tRNAs (13–16). Polyadenylation by PAP I influences sRNA stability, as described for GlmY and RyhB, which are involved in the synthesis of glucosamine-6-phosphate and the expression of the *Fur* regulon, respectively (15, 17, 18). Polyadenylation is also involved in mRNA decay, with a number of transcripts (*rpsO*, *rpsT*, *lpp*, *ompA*, *rmf*, and *trxA*) (19–23) and mRNAs involved in hyperosmotic stress (*osmY* and *otsA*) (24) and carbon metabolism (*ptsG* and *malT*) (15) becoming more stable in the absence of PAP I. Some mRNAs, such as *rne* and *pnp*, which code for RNase E and PNPase, respectively, are destabilized upon PAP I overexpression (25). Although these examples clearly demonstrate that PAP I is involved in regulating the stability of several RNA species, the full scope of the effect of PAP I-mediated polyadenylation on RNA stability has not yet been determined because changes in RNA stability in response to variations in PAP I activity have never been measured across an entire genome.

At the physiological level, RNA stability depends on growth conditions: overall, mRNA stability is negatively correlated with growth rates (26) and is reduced in the stationary phase (27). Our hypothesis is that the stabilization of mRNAs under slow or no growth could be an energy-saving mechanism. This hypothesis is supported by the fact that simultaneous RNA stabilization and ATP decrease have been observed in carbon-starved cells and in cells exposed to sodium fluoride (28, 29). However, the causal link between low ATP levels and RNA stabilization has not yet been established. To our knowledge, the only existing study clearly linking RNA stability and energy status is that of Vargas-Blanco et al.‘s work on *Mycobacterium smegmatis* (30). Understanding the relationship between energy and RNA stability is a major research objective in bacterial physiology. In particular, the role of ATP-dependent enzymes involved in RNA degradation remains to be elucidated, notably that of PAP I involved in RNA polyadenylation and ATP consumption and therefore sited at the crossroads of energy and RNA metabolisms in *E. coli*.

This article reports the first genome-scale analysis of the effect of PAP I activity on the stability of *E. coli* RNAs. We used a strain with an inactivated *pcnB* gene encoding PAP I to reduce the level of genome-wide polyadenylation (15). We compared the half-lives of 2,627 RNAs in the *pcnB* mutant with those measured in the parent strain grown on glucose. The absence of PAP I had a stabilizing effect on *E. coli* RNAs overall. Because PAP I is an ATP-consuming enzyme, we investigated whether PAP I inactivation was related to changes in intracellular ATP levels and, conversely, whether RNA-stabilizing effects could be related to changes in ATP levels. Our results provide the first evidence in *E. coli* of a causal link between ATP levels and RNA stabilization and demonstrate that this is mediated in some cases by PAP I.

## RESULTS

### PAP I inactivation increases overall RNA stability in *E. coli*

**Fig 1:**
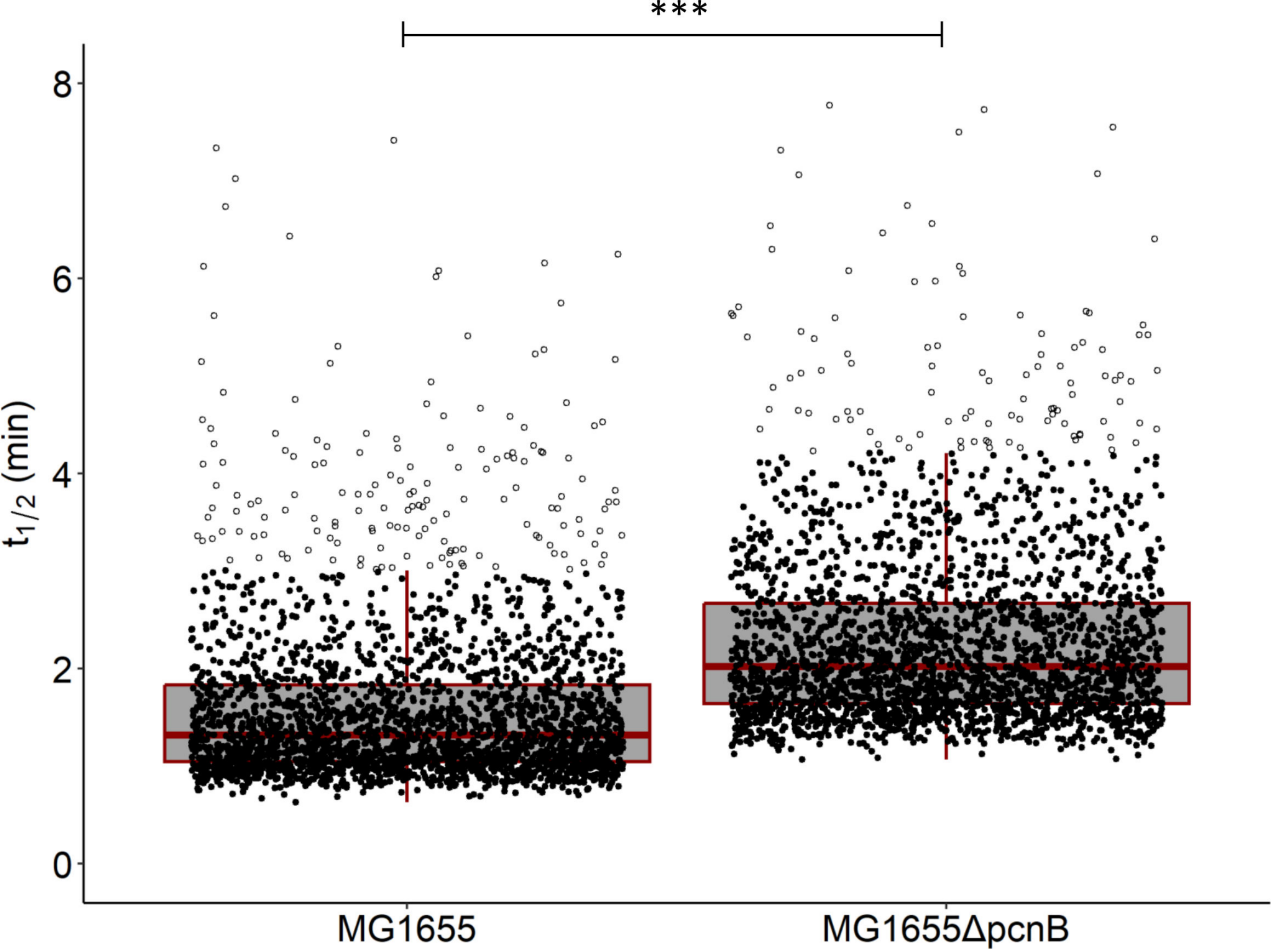
Genome-wide RNA stabilization induced by PAP I inactivation. Box plots of RNA half-lives in *E. coli* MG1655 and MG1655Δ*pcnB* for 2,627 RNAs with reliably determined half-lives in both strains. MG1655 and MG1655Δ*pcnB* cells were exponentially growing in M9 glucose in the bioreactors. Data points on box plots are shown by black filled circles, and outliers are represented by black open circles. Wilcoxon–Mann–Whitney test, ***, *P* < 0.001.

MG1655 and MG1655Δ*pcnB* strains were grown exponentially in M9 glucose. The maximal growth rate was reduced in the absence of PAP I (0.57 ± 0.04 h^−1^ for MG1655Δ*pcnB* versus 0.70 ± 0.02 h^−1^ for MG1655). The RNA half-lives were measured across the entire genome after inhibiting transcription with rifampicin by RNA-Seq analysis at different time points. Half-lives were determined with a classical linear model fitted on the log concentration of RNA. Reliable half-life values were obtained in both strains for 2,627 RNAs. These half-lives were significantly higher on average in the *pcnB* mutant than in the parent strain, with a median value of 2.0 min in MG1655Δ*pcnB* versus 1.3 min in MG1655 (Fig. 1; Wilcoxon–Mann–Whitney test *P*-value < 2.2E^−6^). To account for a potential delay in the onset of exponential decay due to the last round of transcription elongation after rifampicin addition (27) and a possible residual RNA concentration at the end of the decay process (31), we also calculated the RNA half-life by introducing a delay before exponential decay with and without a residual baseline (Fig. S1). With a median delay of 30 s and a median residual concentration of less than 1.5% of the concentration at T0, the introduction of delay and residual baseline decreased the half-life of only some RNAs for MG1655 and MG1655Δ*pcnB* while allowing significant correlations between reliable half-lives calculated with and without delay (Figure S1A, R^2^ > 0.8) and with and without delay plus a residual baseline (Figure S1B, R^2^ > 0.5). The ratios of the half-lives obtained with delay alone (Fig. S1A) and with delay plus residual baseline (Fig. S1B) are similar to that obtained without delay or residual RNA concentration (Fig. 1), indicating that inactivation of PAP I stabilizes RNAs in *E. coli*, irrespective of the half-life calculation method. We then used the half-lives calculated with the classic linear model without the delay and the residual concentration for the rest of the study.

**Fig 2:**
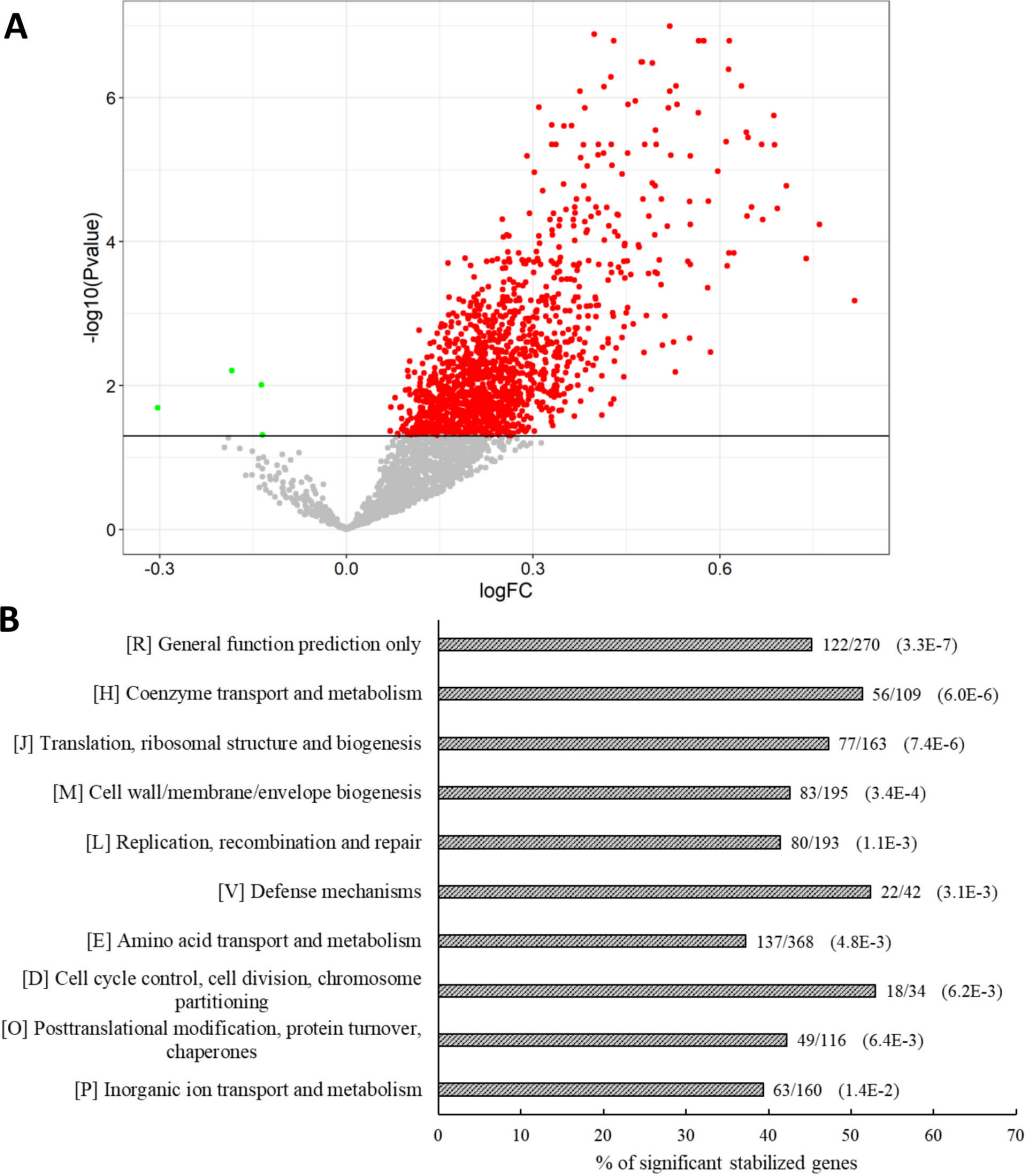
Differences in RNA half-lives between MG1655Δ*pcnB* and MG1655 and functional enrichment. (**A**) Volcano plot of the log2 fold change (logFC) of RNA half-lives between MG1655Δ*pcnB* and MG1655. The values above the horizontal black line correspond to significant differences in fold change (*P* ≤ 0.05). The 1,403 RNAs that were stabilized in the absence of PAP I are shown in red, and the four RNAs that were destabilized are shown in green. The 1,220 RNAs with non-significant differences in stability are shown in gray. Semilogarithmic plots illustrating the degradation profiles of the stabilized and destabilized RNAs between MG1655 and MG1655Δ*pcnB* are shown in Fig. S2. (**B**) Functional enrichment in the 1,403 stabilized RNAs in MG1655Δ*pcnB*. For each cluster of orthologous groups of proteins, the bar represents the percentage of significantly stabilized RNAs relative to the total number of genes in that category. The number of significantly stabilized RNAs and the total number of genes in each category are shown to the right of each bar, with the associated *P* value in parentheses.

Half-life differences were significantly increased for 1,403 individual RNAs in the absence of PAP I, whereas only four were destabilized (Fig. 2A), confirming the destabilizing role of PAP I at the level of individual RNAs. Analysis of RNA-Seq data showed that, depending on the RNA, full-length molecules or fragments were stabilized in the *pcnB* mutant (Fig. S3). The accumulation of *zapB* fragments in MG1655Δ*pcnB* was confirmed by northern blot experiments (Fig. S4A). Although we are aware that stabilized fragments can be non-functional, we still performed a functional analysis of stabilized RNAs. Based on a cluster of orthologous groups of proteins (COG) functional enrichment analysis, the stabilized transcripts are involved in essential biological processes, such as DNA replication, recombination, and repair (80 RNAs), translation, ribosomal structure, biogenesis (77 RNAs), posttranslational modification, protein turnover, chaperones (49 RNAs), wall, membrane and envelope biogenesis (83 RNAs), and cell cycle and division (18 RNAs), and in coenzyme, amino acid, and inorganic ion transport and metabolism (256 RNAs) (Fig. 2B). In addition, the *fli*, *flh*, and *flg* regulons involved in the flagellum assembly were also more stable in the absence of PAP I (Table S1). Stabilization was likewise observed for several transcripts of the RNA degradation machinery, including those encoding RNase E, RhlB, PNPase, RNase R, RNase G, RNase II, and RppH and those coding for RNA-binding proteins, such as Hfq and Rho (Table S1). Many of the stabilized transcripts are involved in defense mechanisms (22 RNAs) in response to environmental stress, including envelope stress (*rcsD* and *rcsB*), osmolarity stress (*envZ* and *ompR*), as well as limitation in oxygen (*arcA* and *arcB*), phosphate (*phoA* and *phoB*), Mg^2+^ (*phoP*), K^+^ (*kdpD*), and nitrogen (*glnL*, *glnG*, *glnD* and *glnB*) or general stress factors, *rpoE* and *rpoH* (Table S1). The RNA levels before rifampicin addition indicated that only 164 of the 1,403 stabilized transcripts had up-regulated expression (Table S1). Analysis of the functional enrichment of the 164 RNAs pointed out four significantly enriched categories: defense mechanisms against specific stresses (8 RNAs, *P* = 1.0E^−4^), cell wall, membrane, and envelope biogenesis (15 RNAs, *P* = 4.4E^−3^), coenzyme transport and metabolism (10 RNAs, *P* = 5.8E^−3^), and general function prediction only (18 RNAs, *P* = 8.3E^−3^). In contrast, the expression of general stress factors, such as the stabilized mRNAs, *rpoE* and *rpoH*, and the seven stabilized transcripts linked to the RNA degradation machinery remained unchanged.

Surprisingly, four transcripts, namely, *yabQ, elaA*, *rcsA*, and *cspE,* were destabilized in the absence of PAP I (Fig. 2A; Table S1). The gene *cspE* codes for one of the nine cold shock proteins (CSPs) in *E. coli* and was the only CSP gene destabilized in the absence of PAP I activity. Destabilization of *cspE* was associated with a 56% decrease in its concentration (Table S1).

### Inactivation of PAP I leads to a decrease in ATP levels in *E. coli*

**Fig 3:**
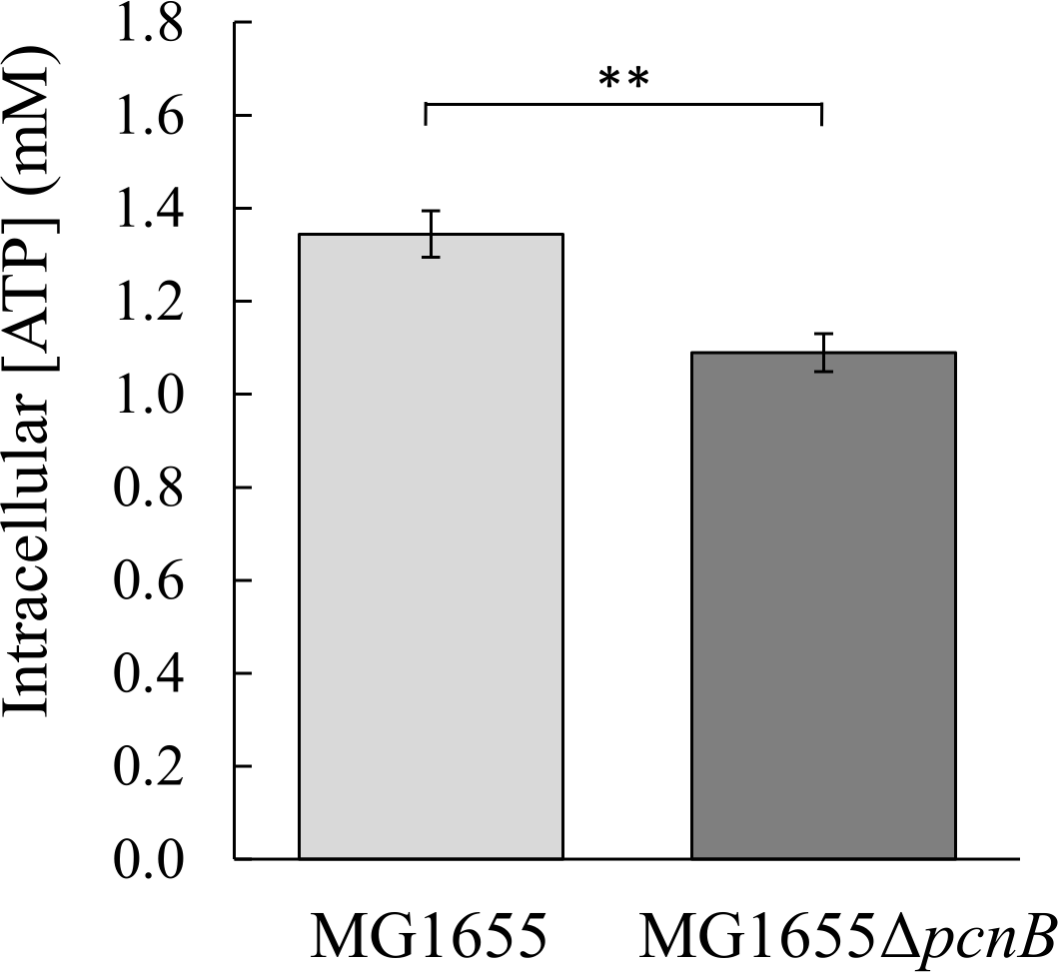
ATP levels. Intracellular ATP concentrations were measured in exponentially growing cells (OD = 1) of MG1655 and MG1655Δ*pcnB* in M9 glucose in flask cultures. **, *P* < 0.01.

Because PAP I is an ATP-dependent enzyme, that is, it consumes one molecule of ATP for each A added to the 3′ end of transcripts, we wondered whether inactivation of PAP I might affect intracellular ATP levels. The ATP levels were measured using a bioluminescence assay in exponentially growing MG1655 and MG1655Δ*pcnB* cells in M9 glucose (Fig. 3). Intracellular ATP levels were significantly (19%) lower in the absence of PAP I (1.09 mM in MG1655Δ*pcnB* versus 1.34 mM in MG1655, *P* = 2.2E^−3^).

### *E. coli* RNAs are stabilized at low ATP levels

**Fig 4:**
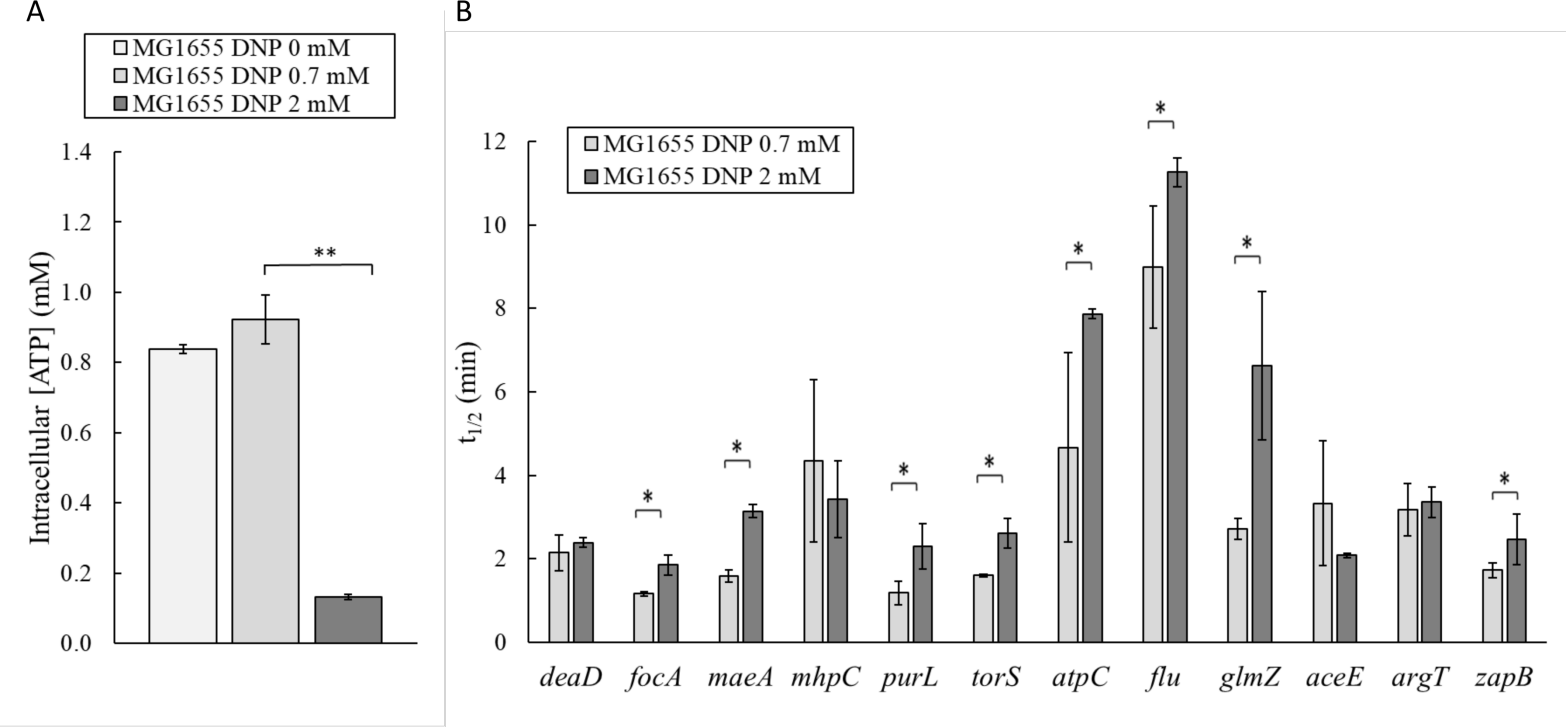
RNA stabilization at low ATP levels induced by treatment with DNP in *E. coli* cells. (**A**) ATP levels in the absence of DNP and in cells treated with 0.7 mM and 2 mM DNP. (**B**) RNA half-lives. MG1655 cells were grown in M9 succinate in flask cultures. Succinate was used instead of glucose to make the DNP-sensitive respiratory chain the main pathway for the ATP synthesis. RNA half-lives were measured by quantitative RT-PCR in cells at high (cells treated with 0.7 mM DNP) and low (cells treated with 2 mM DNP) ATP levels. Representative graphs of the quantification cycle (Cq) versus time illustrating the RNA degradation profiles are shown in Fig. S5A. *, *P* < 0.05; **, *P* < 0.01.

Based on the recent observation of RNA stabilization at low energy levels in *M. smegmatis* (30), we wondered whether this might also occur in *E. coli*, in which case, the overall stabilization observed in the *pcnB* mutant might also be related to a decrease in the energy levels. To address this question, we first studied the link between RNA stability and energy levels in the MG1655 strain by changing the ATP level and measuring how this affected the stability of selected RNAs. We adapted the protocol developed for *M. smegmatis* (30) for *E. coli* growing on succinate as the sole carbon source. Succinate was used instead of glucose to make the respiratory chain the main pathway of ATP synthesis (i.e., to ensure ATP was no longer produced by phosphorylation of the glycolytic substrate) and thus maximize the decrease in the intracellular ATP level when the respiratory chain was inactivated using DNP (32–34). Briefly, we co-treated exponentially growing *E. coli* MG1655 cells with rifampicin and DNP, an ATP uncoupler (32). We determined the half-lives by quantitative real-time RT-PCR of 12 RNAs at high (0.7 mM DNP treatment) and very low (2 mM DNP treatment) intracellular ATP levels. The selected RNAs differ in CDS length, concentration, stability, genetic environment, function, and protein localization and are, therefore, representative of the range of RNAs present in *E. coli* (Table 1). Treatment with 0.7 mM DNP did not affect the level of intracellular ATP in MG1655 (around 0.9 mM ATP concentration in the absence of DNP and in cells treated with 0.7 mM), while treatment with 2 mM reduced it by 86% to 0.13 mM (Fig. 4A). Eight (*focA*, *maeA*, *purL*, *torS*, *atpC*, *flu,* GlmZ, and *zapB*) of the 12 selected RNAs were significantly stabilized when the ATP level was reduced, and none was significantly destabilized (Fig. 4B). The stabilized RNAs had different concentrations and sizes (Table 1), indicating that these two factors known to influence RNA stability (35, 36) were not involved in this case. We also sequenced the total RNA samples corresponding to 0.7 mM and 2 mM DNP. Based on the RNA-Seq data of the eight stabilized RNAs, we found that, depending on the RNA, full-length transcripts or RNA fragments were stabilized at low ATP levels (Fig. S6). Northern blot experiments confirmed the absence of *zapB* RNA fragments stabilized at low ATP levels (Fig. S4B). Altogether, these results demonstrate that a sharp decrease in ATP levels induces RNA stabilization for most of our selected RNAs. This stabilization was also extended to many other RNAs. We determined genome-wide RNA half-lives from RNA-Seq data and found that 1,769 RNAs were stabilized between 0.7 mM and 2 mM DNP (Fig. S7). The RNA stabilization observed at low ATP levels is likely due to the lower activity of ATP-dependent enzymes, which could act directly on RNA stability as part of the RNA degradation machinery or indirectly via other cellular processes.

**TABLE 1:** List of RNAs selected for half-life measurements by RT-qPCR^*a*^

Gene name	Gene number	Protein/RNA function	COG	CDS length (bp)	Operon	RNA concentration (AU)	*t*_1/2_ (min)	Protein cellular localization
*deaD*	b3162	ATP-dependent RNA helicase DeaD	Translation, ribosomal structure and biogenesis	1890	Yes	87	2.1	Cytosol
*focA*	b0904	Formate channel transporter FocA	Inorganic ion transport and metabolism	858	Yes	11	1.7	Inner membrane
*maeA*	b1479	Malate dehydrogenase	Energy production and conversion	1698	No	61	2.4	Cytosol
*mhpC*	b0349	2-hydroxy-6-oxononatrienedioate hydrolase	NA	867	Yes	1	3.4	Cytosol
*purL*	b2557	Phosphoribosylformylglycinamide synthetase	Nucleotide transport and metabolism	3888	No	195	1.1	Cytosol
*torS*	b0993	Sensor histidine kinase TorS	Signal transduction mechanisms	2745	No	2	1.5	Inner membrane
*atpC*	b3731	ATP synthase F_1_ complex subunit ε	Energy production and conversion	420	Yes	244	NA	Inner membrane
*flu*	b2000	Antigen 43 (Ag43) autotransporter	NA	3120	No	21	NA	Outer membrane
*glmZ*	b4456	small regulatory RNA GlmZ	NA	172	No	77	NA	NA
*aceE*	b0114	Pyruvate dehydrogenase	Energy production and conversion	2664	Yes	494	2.3	Cytosol
*argT*	b2310	Lysine/arginine/ornithine ABC transporter periplasmic binding protein	Amino acid transport and metabolism	783	Yes	31	2.6	Periplasmic space
*zapB*	b3928	Cell division factor ZapB (Z-ring)	Function unknown	246	No	50	1.7	Cytosol

^*a*^ COG: clusters of orthologous groups. NA: not available. RNA concentrations and half-lives (*t*_1/2_) are those measured in strain MG1655 by RNA-Seq in the omics study.

### Contribution of PAP I to RNA stabilization at low ATP levels

**Fig 5:**
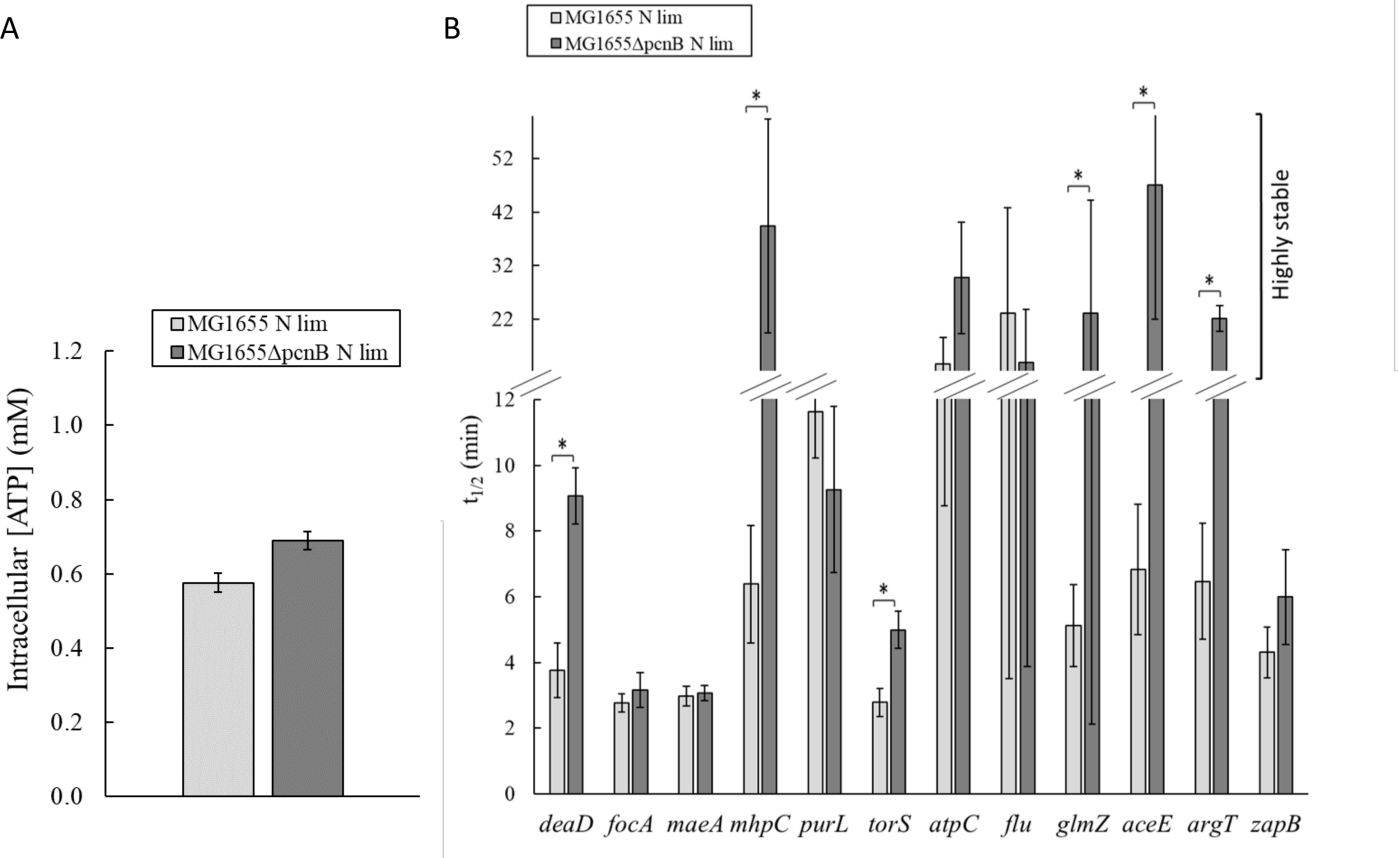
ATP levels and RNA half-lives in MG1655 and MG1655Δ*pcnB* under nitrogen limitation. (**A**) ATP levels. (**B**) RNA half-lives were measured by quantitative RT-PCR. Representative graphs of the quantification cycle (Cq) versus time illustrating the RNA degradation profiles are shown in Fig. S5B. Half-lives longer than 12 min were classified as highly stable. Cells were grown in M9 glucose limited in NH_4_Cl to obtain similar intracellular concentrations of ATP in the two strains and saturate all ATP-dependent enzymes. *, *P* < 0.05.

We then investigated whether PAP I might be involved in the RNA stabilization observed at low ATP levels. One explanation for this could be that ATP-consuming enzymes, notably PAP I, are inactivated by the low ATP concentrations. To specifically study the involvement of PAP I independently of any changes in the activity of other ATP-dependent enzymes in the RNA degradation machinery, we grew *E. coli* cells with high levels of ATP to saturate all ATP-dependent enzymes and then searched for RNAs that were stabilized in the absence of PAP I. The underlying idea is that RNAs stabilized in the absence of the PAP I enzyme (all other ATP-dependent enzymes being present and functional at high ATP levels) correspond to RNAs that are also stabilized at low ATP levels when PAP I activity is reduced due to low substrate availability. MG1655 and MG1655Δ*pcnB* cells with the required intracellular ATP levels (around 0.6 mM) were obtained by nitrogen-limited growth (Fig. 5A). We then compared the half-lives measured by quantitative RT-PCR of the 12 selected RNAs in MG1655 and MG1655Δ*pcnB* at similarly high intracellular ATP levels (Fig. 5B). Six RNAs (*deaD*, *mhpC*, *torS,* GlmZ, *aceE*, and *argT*) were more stable in MG1655Δ*pcnB* than in MG1655, demonstrating that the stability of these RNAs is dependent on PAP I activity. Among these RNAs, *torS* and GlmZ were also stabilized at low ATP levels in MG1655 (see above). Consequently, we can conclude that these RNAs, which are stabilized when only the PAP I enzyme is absent, were probably also stabilized when MG1655 was exposed to low ATP levels due to a limitation of PAP I activity by the availability of ATP.

**Fig 6:**
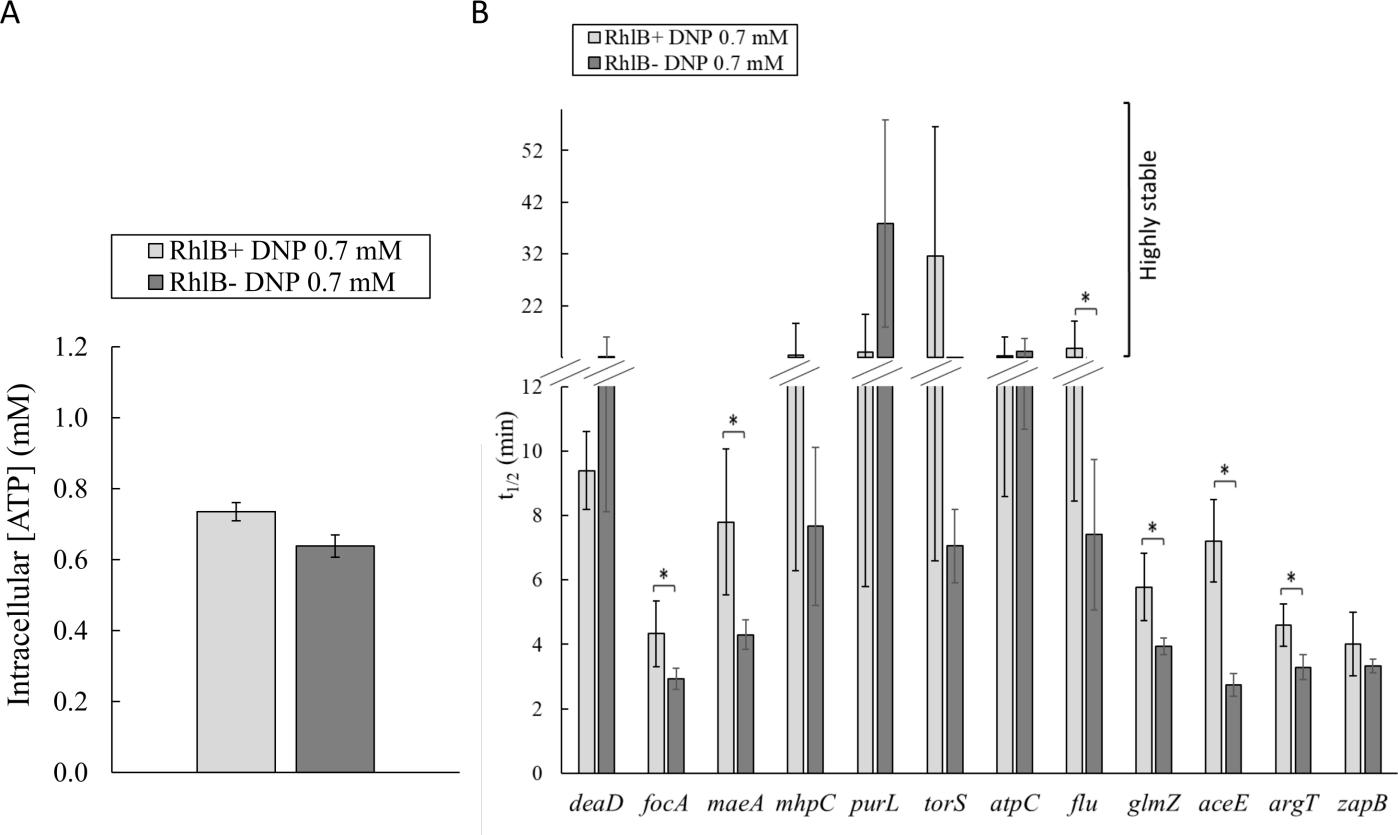
ATP levels and RNA half-lives in the presence or absence of RhlB. (**A**) ATP levels. (**B**) RNA half-lives were measured by quantitative RT-PCR. Representative graphs of the quantification cycle (Cq) versus time illustrating the RNA degradation profiles are shown in Fig. S5C. Cells were grown in M9 succinate in flask cultures treated with 0.7 mM DNP. Succinate was used instead of glucose to make the DNP-sensitive respiratory chain the main pathway for ATP synthesis. Half-lives longer than 12 min were classified as highly stable. *, *P* < 0.05.

In contrast, six other RNAs (*focA, maeA, purL, atpC, flu*, and *zapB)* had similar half-lives in MG1655Δ*pcnB* and MG1655 at high intracellular ATP levels (Fig. 5B), indicating that they are not regulated by PAP I. However, since these RNAs were stabilized at low ATP levels in MG1655, we wondered whether this could be due to the inactivation of RhlB, which was another ATP-consuming enzyme in the RNA degradation machinery. To test this hypothesis, the half-lives of *focA*, *maeA*, *purL*, *atpC*, *flu*, and *zapB* RNAs were measured by quantitative RT-PCR and compared at high ATP levels to saturate all ATP-dependent enzymes in the presence and absence of RhlB (Fig. 6). At around 0.7 mM ATP (obtained by adding 0.7 mM of DNP), none of the RNAs were more stable in the RhlB^−^ strain than in the RhlB^+^ strain, indicating that the stability of these six RNAs does not depend on RhlB activity. Altogether, these results show that the stability of *focA*, *maeA*, *purL*, *atpC*, *flu*, and *zapB* RNAs does not vary in the absence of PAP I or RhlB. Their stabilization at low ATP levels in MG1655 must, therefore, be due to the reduced activity of another ATP-dependent enzyme, part of the RNA degradation machinery (see Discussion) or involved in other cellular processes affecting RNA stability, such as translation.

### Correlation between ATP levels and RNA stability depends on ATP levels

**Fig 7:**
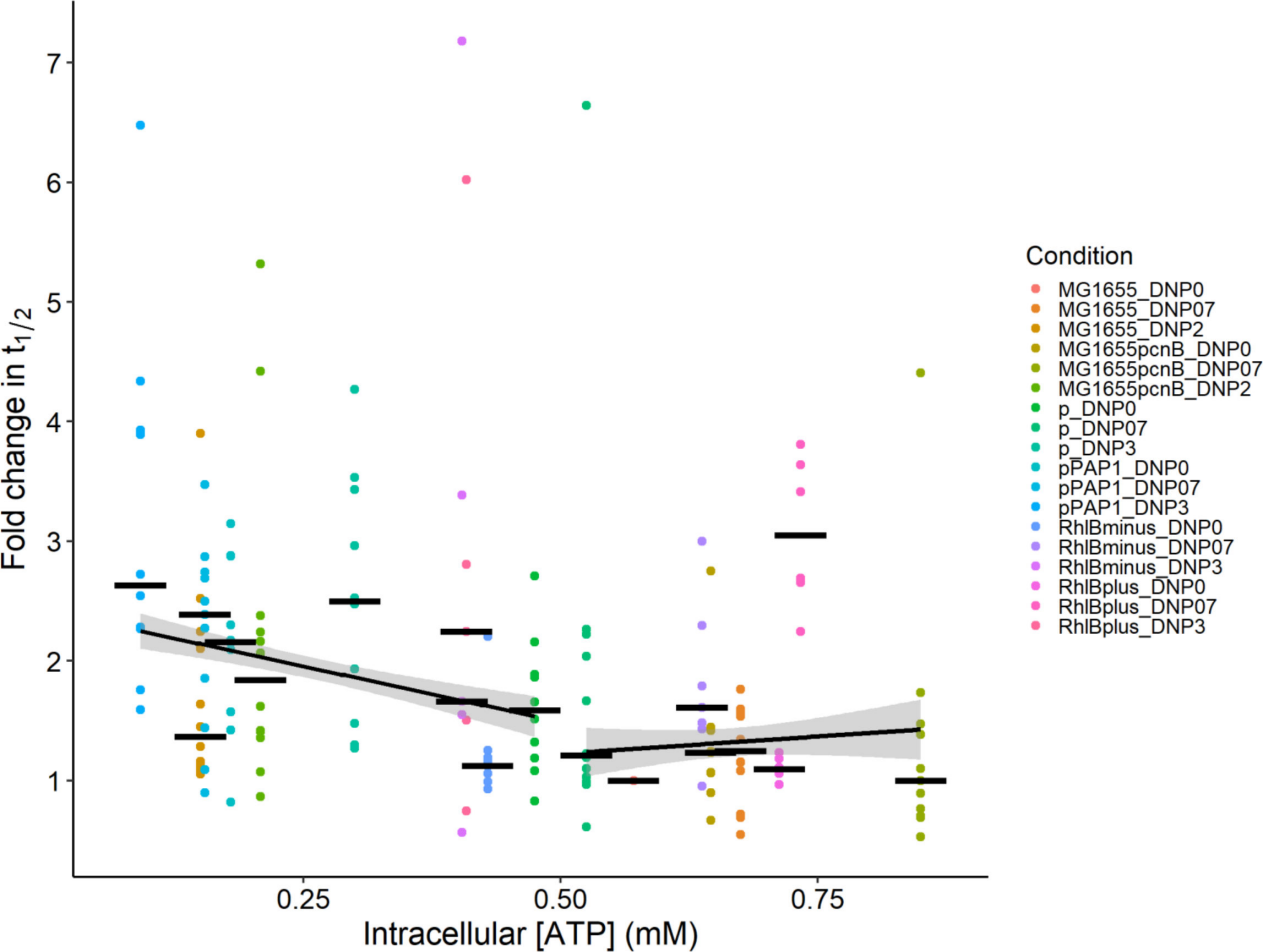
Correlation between ATP levels and RNA stability. ATP levels and the half-lives of 12 selected RNAs were performed in the presence of no, low (0.7 mM), and high (2–3 mM) concentrations of DNP in M9 succinate for strains MG1655, MG1655Δ*pcnB*, RhlB^+^, RhlB^−^, MG1655Δ*pcnB*/p, and MG1655Δ*pcnB*/pPAP I (values in Table S3). The half-life of each RNA was measured by quantitative RT-PCR and expressed as a fold change relative to the value in MG1655 grown without DNP. Representative graphs of the quantification cycle (Cq) versus time illustrating the RNA degradation profiles are shown in Fig. S5. Data points are colored by condition, and the 18 conditions are listed on the figure to specify the strains and DNP treatments used. The median value of all the points in a given condition is indicated by a horizontal black bar. Segmented linear regressions on median values are shown in black, and the associated standard error ribbons are depicted in gray. The difference in slope (a slope value of −1.9 for ATP levels < 0.5 mM *versus* a slope value of 0.5 for ATP levels > 0.5 mM) was significant (*P* = 3.2E^−3^) using Student’s *t*-test.

To analyze the correlation between ATP levels and RNA stability, we complemented our data on ATP levels and RNA half-lives in DNP-treated strains grown in M9 succinate, with measurements under new conditions to generate a 3 × 6 matrix of three DNP treatments (no, low concentration, and high concentration treatment) and six strains, namely, wild-type MG1655 and five strains with mutations in the RNA degradation machinery (absence of PAP I, inactivation or not of RhlB, and overexpression or not of PAP I; Fig. 7; Table S3). We observed RNA stabilization when ATP levels were reduced, but only for ATP concentrations below 0.5 mM. Above 0.5 mM, changes in ATP levels were not associated with any significant change in RNA stability.

## DISCUSSION

This study is the first genome-wide investigation of the effect of PAP I activity on RNA stability in *E. coli* or any other bacterium. Our results clearly demonstrate that PAP I plays a significant role in regulating RNA stability across the genome as a whole. Absence of PAP I was associated with an overall stabilization of RNAs in *E. coli*, with 1,403 RNAs becoming more stable and only four becoming less stable. Enzymes of the degradation machinery other than PAP I should not be involved in this overall RNA stabilization because of their constant gene expression. We found that depending on the RNA, full-length molecules or decay fragments were stabilized in absence of PAP I. This confirms that PAP I polyadenylation can affect both decay intermediates and full-length transcripts (15, 37). Full-length transcripts may accumulate because of a slowdown in the initiation of their degradation triggered by 3′ exoribonucleases. Decay fragment stabilization may occur because of a slower turnover when RNA degradation is initiated by endoribonucleolytic cleavage. Decay fragments can be non-functional and therefore probably have less impact on gene expression than the stabilization of functional full-length transcripts. The effect of the type of stabilized RNA molecules on the regulation of gene expression and on the physiology of *E. coli* cells in the absence of PAP I could be studied more closely, gene by gene, using proteomic approaches.

Functional analysis of the stabilized transcripts showed that they are involved in essential biological functions (e.g*.,* replication, recombination, and DNA repair and translation). These stabilized transcripts included members of several two-component systems, including systems involved in specific environmental stress response but not in the general stress response. We, therefore, ruled out a global stabilization of RNAs due to the general response to stress. Inactivation of PAP I also stabilized transcripts involved in flagellum assembly. These effects could be related to the phenotypic features of the *pcnB* mutant, such as its different sensitivity to stress (15, 24, 38, 39) and decreased motility on soft agar (39). These results highlight the fact that PAP I polyadenylation regulates the stability of transcripts involved in a broad range of biological functions in *E. coli*, with a potentially major impact on cell physiology.

We found that global RNA stabilization in MG1655Δ*pcnB* grown on glucose was associated with almost 20% reductions in growth rate and ATP levels. We can rule out that the overall stabilization of the RNAs is the result of the reduction in growth rate in the *pcnB* mutant. RNA stabilization as a function of growth rate is indeed low when the growth rate remains above 0.4 h^−1^ (26), which is the case for the two strains, MG1655 and MG1655Δ*pcnB*, with 0.7 and 0.57 h^−1^, respectively. We also identified only a very limited number of RNAs (*glyS, fruB, glyQ, cysK, yqjH, tnaA, ulaE, prfB*, and *fabA*) stabilized in MG1655Δ*pcnB* and also stabilized when the growth rate decreased from 0.63 to 0.4 h^−1^ (26). Conversely, RNA stabilization can lead to a reduction in growth by reduced release of ribonucleotides when RNA degradation is affected in the *pcnB* mutant, which could limit the synthesis of deoxyribonucleotides and, in turn, DNA synthesis and growth (40). The decrease in ATP levels in MG1655Δ*pcnB* is rather surprising because, in the absence of PAP I, ATP is no longer consumed by the polyadenylation process, and the reduced growth rate may indicate a reduction in growth-related energy demand. Given that several di/triphosphate nucleotide synthesis and consumption pathways co-exist in *E. coli* (KEGG Database, eco00230 for purine metabolism and eco00240 for pyrimidine metabolism), further experiments to compare nucleotide pools and intracellular energy fluxes in the presence and absence of PAP I are required to fully understand the interaction between RNA polyadenylation, ATP levels, and growth rates.

Data in the literature suggest a potential connection between energy status/growth rate and RNA stability regulation in *E. coli* cells (26–29). Our analyses of a selection of RNAs and of the whole RNA population after DNP treatment provide the first evidence of RNA stabilization induced by a sharp decrease in ATP levels in *E. coli* MG1655, with a clear causal link between low ATP levels and RNA stabilization. This causal link has only previously been established in *M. smegmatis* cells (30). In our measurements of ATP levels and RNA half-lives in MG1655 and strains with mutations in the RNA degradation machinery, the stabilizing effect of low ATP levels was only observed below 0.5 mM. This implies that the RNA stabilization observed in the omics study of MG1655Δ*pcnB* at an ATP concentration above 1 mM was not due to any ATP-level effect on the activity of the RNA degradation machinery.

The ATP level threshold, below which RNA stabilization was observed, that is, 0.5 mM, is very close to the Km range reported for PAP I (0.13 mM) and the RNA helicase DbpA (0.33 mM) (41, 42). RNA stabilization at low ATP levels (e.g*.*, after 2 mM DNP treatment during growth on succinate) may, thus, be explained by a decrease in the activity of PAP I and RhlB, two ATP-consuming enzymes involved in RNA degradation, due to low substrate availability. We found several RNAs whose stability is indeed PAP I-dependent, including *torS* and GlmZ RNAs, which are destabilized by PAP I. The stability of GlmZ sRNA was already known to be indirectly regulated by PAP I through the polyadenylation of GlmY sRNA (17). The effect of PAP I on *torS* mRNA does not seem to have been reported before, and it is not clear whether this is a direct or an indirect effect. We also identified six RNAs (*focA*, *maeA*, *purL*, *atpC*, *flu*, and *zapB*) whose stabilization at low ATP levels did not depend on PAP I activity. For these RNAs, we also excluded a stabilizing effect of RhlB inactivation at low ATP levels. However, we cannot exclude a possible effect of RhlB inactivation on other RNAs that were not investigated here. Although our results indicate that the stabilization of the six RNAs is not related to an ATP effect via substrate limitation of PAP I and RhlB activity, low ATP levels may affect the activity of other enzymes in the RNA degradation machinery: ATP may directly act on RNases as an effector (as with RNase R, which binds but does not hydrolyze ATP for helicase activity *in vitro* [43]) or indirectly via still unknown mechanisms.

At the physiological level, under energy-limited conditions, RNA stabilization could be a means of saving energy by reducing RNA turnover. RNA turnover involves three energy-demanding steps: RNA synthesis, RNA degradation, and regeneration of nucleoside triphosphates from the nucleoside mono- or diphosphates released by degradation. Our study provides evidence that PAP I is involved in reducing RNA turnover at low ATP levels by inactivation-induced stabilization of certain RNAs. This result illustrates how the RNA polyadenylation activity of PAP I links the regulation of RNA stability to energy status in *E. coli*.

## MATERIALS AND METHODS

### Strains and media

The strains and plasmids used in this study are listed in Table 2. We used *E. coli* MG1655 and its derivative MG1655Δ*pcnB*, carrying an inactivated *pcnB* gene, which encodes PAP I (39). PAP I overexpression was obtained by transforming MG1655Δ*pcnB* with the pBMK11 plasmid, carrying *pcnB* under an IPTG-inducible P_lac_ promoter or the empty plasmid pBMK14 as a control (25). These strains are referred to as MG1655Δ*pcnB*/pPAP I and MG1655Δ*pcnB*/p, respectively. RhlB was inactivated by the point mutation DQAD in the DEAD box. First, DNA fragments with appropriate 3′ and 5′ extensions synthesized by PCR using genomic DNA as template were inserted into the chromosome of NCM3416 by λ Red recombination (44). The coding sequence extending from the DEAD-box to the 3′ end of the *rhlB* gene (residues 166–421) was deleted by insertion of a *cat* cassette (from pKD3) into NCM3416 to create strain SAJ102. Second, the *rhlB* deletion was repaired in strain SAJ103 by insertion of a *rhlB(DQAD)-kan* DNA fragment that was synthesized by cross-over PCR. The construct was validated by PCR of genomic DNA and sequencing. The *kan* gene (from pKD4), which lacks a promoter and flanking *frt* sites, is co-transcribed with *rhlB*. The genomic fragment containing the *kan* gene was amplified from SAJ103 and used to transform NCM3416 carrying plasmid pKD46 to obtain strain SCR101, with the *kan* gene right after the *rhlB* gene and an intact DEAD box. RhlB^+^ and RhlB^−^ were then constructed in MG1655 by P1-mediated transduction from SCR101 and SAJ103, respectively. Transductants were selected with kanamycin and confirmed by PCR and sequencing. DNA primers are listed in Table S2.

**TABLE 2:** List of the strains and plasmids used in this study

Strain or plasmid	Genotype or features	Source or reference
Strains		
MG1655	*E. coli* K-12, F−, λ−, *rph-1*	CGSC
MG1655Δ*pcnB*	MG1655, Δ*pcnB::kan*	(39)
NCM3416	*E. coli* K12, F−, λ−, *rph*+, *zib*-207::Tn10	(45)
SAJ102	NCM3416, *rhlBΔ(166-421)::cat*	This work
SAJ103	NCM3416, *rhlB(DQAD)− kan*	This work
SCR101	NCM3416, *rhlB(DEAD)− kan*	This work
RhlB^+^	MG1655, *rhlB(DEAD)− kan*	This work
RhlB^−^	MG1655, *rhlB(DQAD)− kan*	This work
Plasmids		
pBMK11	Carries *pcnB* under the IPTG-inducible *lac* promoter	(25)
pBMK14	Same as pBMK11 but without *pcnB*	(25)
pKD3	Chloramphenicol resistance	(44)
pKD4	Kanamycin resistance	(44)
pKD46	ts origin, ampicillin resistance, λ Red recombinase	(44)

The strains were grown in M9 minimal medium, as previously described (26). M9 medium was supplemented with 16.7 mM glucose (M9 glucose) or 85 mM of succinate (M9 succinate). The medium was supplemented with chloramphenicol (20 µg/mL) and IPTG (1 mM) if required. Nitrogen limitation was achieved by 6 h of culture in M9 glucose medium containing a six-fold reduced NH_4_Cl concentration (0.34 g/L).

### Growth conditions on glucose

After overnight precultures in M9 glucose, cells were washed twice with NaCl 9 g/L and used to inoculate 1.8 L of M9 glucose at OD_600_= 0.1 in a Biostat B+ bioreactor. Temperature was regulated at 37°C and pH at 7, and dissolved oxygen pressure was set to a minimum of 30%. When OD_600_ reached 1.2, samples were collected for transcriptomic analysis; this time point was defined as the reference time point (T0) for half-life measurements. Rifampicin (0.5 g/L) was then added to the culture to inhibit transcription initiation, and cells were sampled at different times and frozen in liquid nitrogen. These experiments were repeated thrice on independent cultures for each strain.

### Growth conditions on succinate and treatments with rifampicin and DNP

After overnight growth in M9 glucose, cells were washed with M9 succinate and used to inoculate Erlenmeyer flasks with M9 succinate supplemented with chloramphenicol and IPTG if required at OD_600nm_= 0.1. At OD_600nm_= 0.8, rifampicin (0.5 g/L) was added, and cell samples were taken over time and rapidly frozen in liquid nitrogen. When required, cells were treated with either 0.7, 2, or 3 mM 2,4-dinitrophenol (DNP). Rifampicin and DNP were added simultaneously for MG1655, MG1655Δ*pcnB*, M1655pcnB/p, and MG1655Δ*pcnB*/pPAP I. Rifampicin was added 15 min after DNP treatment for RhlB^+^ and RhlB^−^ to allow ATP levels to decrease. All experiments were performed in biological duplicates and technical triplicates for all strains.

### RNA extraction

For experiments on glucose, after thawing and centrifugation, the cells were suspended in Tris–EDTA buffer and lysed mechanically with glass beads in the presence of phenol. Total RNA was extracted with TRI Reagent (Sigma Aldrich) from 12 samples from the three biological replicates as follows: for MG1655, T0, 0.5, 2 and 4 min for the first replicate; T0, 1, 2.5 and 5 min for the second replicate; and T0, 1.5, 3 and 7 min for the third replicate; for the *pcnB* mutant, T0, 1, 3 and 7 min for the first replicate; T0, 0.5, 1.5 and 5 min for the second replicate; and T0, 2, 4 and 11 min for the third replicate. DNA contamination was removed with Turbo DNase treatment (Invitrogen). For experiments on succinate, after mechanical lysis with glass beads, total RNA was extracted using RNeasy Mini Kit (Qiagen) at times T0, 0.4, 1, 2, 4, and 7 min for the two biological replicates. DNA contamination was removed by DNase I treatment.

### RNA-Seq

For experiments on glucose, total RNA was ribodepleted using riboPOOLs Kit (siTOOLs Biotech). Subsequently, 10 ng ribodepleted RNA was further used to construct a sequencing library using Ion Total RNA-Seq Kit v2 and Ion Xpres RNA-Seq Barcode Kit (Thermo Fisher Scientific). The library was constructed according to the manufacturer’s protocol. Next, 15 pg/µL of each library was amplified by emulsion PCR on Ion One Touch 2 instrument. Then, a 100 base pair (bp) single-end library was sequenced on Ion GeneStudio S5 System with Ion 540 Chip at the GeT-BioPuces platform (http://get-biopuces.insa-toulouse.fr/). For experiments on succinate, total RNA was ribodepleted using Ribo-Zero Kit (Illumina). A paired-end 150 bp sequencing strategy was performed on Illumina PE150 platform (Novogene). Reads were mapped onto the *E. coli* genome (version NC_000913.3, GenBank) with the tmap routine in Torrent and Bowtie2 for Illumina sequencing. Counting was performed with HTSeq-count for ion torrent sequencing and featureCounts for Illumina sequencing. The linearity range between the number of reads and the RNA concentration was established above 10 reads using Ambion ERCC RNA spikes. To account for a potential effect of the read cutoff values, we compared the numbers of RNAs with reliable half-life, stabilized RNAs, and destabilized RNAs when using read cutoff values of 10 and 100 (Table S4). Increasing the read cutoff value decreased the number of reliable RNA half-lives, but did not substantially alter the proportion of stabilized and destabilized RNAs.

### Transcriptome

For the transcriptome analysis on T0 samples, transcripts with fewer than 10 reads in five of six samples were excluded from the analysis, as were duplicated genes and overlapping genes with at least 50 bp in common. Trimmed mean of M values normalization was applied to correct for experimental variations (46). Differential RNA concentrations were evaluated using an exact test for a difference in means between two groups of negative binomial random variables (46). The *P* values were adjusted for multiple testing using the Benjamini and Hochberg false discovery rate method (47). Differences in RNA concentrations were considered significant at *P* < 0.01.

### Genome-wide determination of RNA half-lives

Genome-wide measurements of RNA half-lives were performed using 12 sets of RNA-sequencing data (T0 samples and time points after rifampicin addition). The sequencing data were normalized using the first three invariant genes in each strain (genes with the lowest variance in rank between sequencing data sets, namely, b2621 (*ssrA*), b2911 (*ssrS*), and b3760 (*aspT*) on glucose and b2621 (*ssrA*), b3123 (*rnpB*), and b2092 (*gatC*) on succinate. The linear regression coefficient (*k*) of log(RNA) = log(*a*_0_) − *k* × *t*, with *a*_0_ as the RNA concentration at *T*_0_, and the associated coefficients of determination (*R*^2^) were calculated for each RNA species. The values obtained for *k* were only considered reliable if the associated *R*^2^ was >70%. RNA half-lives (*t*_1/2_) were obtained from *k* using the relationship *t*_1/2_ = ln2/*k* and compared using *t*-tests. Differences were considered statistically significant if the adjusted *P* value was <0.05. The stabilized and destabilized RNAs in MG1655Δ*pcnB* versus MG1655 and the associated concentrations, are listed in Table S1.

### Estimation of RNA half-lives by quantitative PCR

A 5 µg sample of the total RNA was then retrotranscribed using SuperScript II Reverse Transcriptase and RNase H (Invitrogen), and complementary DNA was purified on MicrospinG-25 (Cytiva). Quantitative PCR was performed with the primer pairs listed in Table S2 using IQTM SYBR Green Supermix (Biorad). Fluorescence was read using LightCycler 480 II (Roche). Each quantification was performed in technical duplicates. Primer specificity was determined on a standard range of genomic *E. coli* MG1655 DNAs and ranged from 85 to 95%. The RNA quality was checked using Bioanalyzer 2100 (Agilent). The linear regression coefficient (*k*′) of *C*_*q*_ (quantification cycle) versus time (six points) and its associated coefficient of determination, *R*^2^, were calculated for each RNA species, for each DNP treatment condition, and for each strain. The values obtained for *k*′ were only considered reliable if the associated *R*^2^ was >50%. RNA half-lives were obtained from *k*′ using the relationship *t*_1/2_= 1/*k*′. RNAs with a half-life greater than 12 min or so stable that the half-life could not be reliably quantified were classified as very stable. Differences in half-lives were tested for statistical significance using Wilcoxon–Mann–Whitney tests on two biological replicates and two technical replicates.

### ATP quantification

ATP levels were determined using BacTiter-Glo Microbial Cell Viability Assay Kit (Promega). After centrifugation of 0.8 mL of culture, the cell pellet was resuspended in 0.8 mL of DNP-supplemented growth medium. Samples (75 µL) were transferred to a black 96-well plate containing 125 µL of a BacTiter Glo reagent, shaken for 10 s, and incubated for 5 min at room temperature. Luminescence was measured using a Synergy H1 Microplate reader (BioTek). ATP quantification using BacTiter-Glo Microbial Cell Viability Assay Kit was validated by ion chromatography–mass spectrometry MS/MS measurements using a protocol described previously (48). Intracellular ATP levels were expressed in mM considering 8 × 10^11^ cells/L at OD_600_ = 1 and an intracellular volume of 3 fL per cell (49). Differences in ATP levels were tested for statistical significance using Wilcoxon–Mann–Whitney tests on two biological replicates and three technical replicates.

### Functional enrichment

Functional categories enriched in transcript subgroups were determined using the hypergeometric test with the COG of proteins database. Enrichments were considered significant at *P* < 0.05.

## Data Availability

Raw and processed RNA-seq data have been deposited in the Gene Expression Omnibus data repository and are accessible with GEO accession number GSE248472 for glucose experiments and with GEO accession number GSE272651 for succinate experiments.

## SUPPLEMENTAL MATERIAL

Supplemental material mbio.02680-24-s0001.pdf Table S1 and S3 captions; Tables S2 and S4; Fig. S1–S7.

Table S1 mbio.02680-24-s0002.xlsx Stabilized and destabilized RNAs in the MG1655ΔpcnB strain.

Table S3 mbio.02680-24-s0003.xlsx ATP levels and the half-lives of 12 selected RNAs.

ASM does not own the copyrights to Supplemental Material that may be linked to, or accessed through, an article. The authors have granted ASM a non-exclusive, world-wide license to publish the Supplemental Material files. Please contact the corresponding author directly for reuse.
