# Supplementary material for: RNA stability is regulated by both RNA polyadenylation and ATP levels, linking RNA and energy metabolisms in Escherichia coli
Source: mBio. 2024 Nov 29;16(1):e02680-24. doi: 10.1128/mbio.02680-24 (PMC11708017; doi:10.1128/mbio.02680-24)
Supplement: Supplemental material — Table S1 and S3 captions; Tables S2 and S4; Fig. S1–S7. [file mbio.02680-24-s0001.pdf]

Supplemental Material

Table S1 (excel file): Stabilized and destabilized RNAs in the MG1655Δ*pcnB* strain. NA: not available, FC: fold change, AU: arbitrary unit. UP/DOWN: RNAs with up-regulated/ down-regulated expressions among the 1,403 stabilized and four destabilized transcripts (for significant P value < 0.01). COGs: clusters of orthologous groups : [J], translation, including ribosome structure and biogenesis; [L], replication, recombination, and repair; [K], transcription; [O], molecular chaperones and related functions; [M], cell wall structure and biogenesis and outer membrane; [N], secretion, motility, and chemotaxis; [T], signal transduction; [P], inorganic ion transport and metabolism; [C], energy production and conversion; [G], carbohydrate metabolism and transport; [E], amino acid metabolism and transport; [F], nucleotide metabolism and transport; [H], coenzyme metabolism; [I], lipid metabolism; [D], cell division and chromosome partitioning; [R], general functional prediction only; and [S], no functional prediction.

Table S2: Primers used in the study.

Primers used in RT-qPCR			
**Gene name**	**Gene number**	**Forward primer sequence (5′--> 3′)**	**Reverse primer sequence (5′--> 3′)**
focA	b0904	ACAGGCACAATGCCCTTCGG	GCCCACTCGCCTTAGCAACAAC
torS	b0993	GATGGCGCTGTTAACCCTGACC	GCTGGCTTCGCTCAACTGGC
maeA	b1479	GCTGGCCCTGTACTGCTGGAAT	CCATGCTCGTTCCGCTTGTTC
purL	b2557	CCGAATCAACAAACTGCTGGCA	GGCGTTCAAGTTGTGCGTGCT
mhpC	b0349	CCGCCACCAGCCGTTTTCT	TTCGCCCAGCCAGTAGCACC
deaD	b3162	GCAGATCTGGGCCTGAAGGCT	CATACCCAGAACGTCGCGGC
flu	b2000	GTGTGGCGGTTGCACTGTCT	TTTGCCAGTGTTCCGCCGTT
aceE	b0114	GCGTCCTTCCAGTCTTCCGC	GGGGAGATGTGGCCCTGGAA
atpC	b3731	GCAATTCGCGGCCAGGATCT	CAGTTCCGCAGACGCCTGAG
argT	b2310	ACGCTAACGAGACCTGGCGT	CGCAGCATCCAGACGTCCTG
zapB	b3928	CCCAGCATCAGCGCGAAGAG	CCTCTTCCATGCGACCCAGC
glmZ	b4456	CGGAATGACGCAGAGCCGTTTA	TGGGTGCTTCACTCAACGTTGT

Primers used to construct the *rhlB* mutant	
**Name**	**Sequence (5′--> 3′)**
OAJ18e	CTCGGTGCCATTCAGGTGGTGGTACTGGACCAGGCCGATCGCATGTACGATCTG
OAJ19e	CTCATAACACCCCTTGTATTACTGTTTTTAACCTGAACGACGACGATTACG
OAJ21s	GATACAGTTTGAATGATTTTGAGTATG
OAJ22e	GATACAGTTTGAATGATTTTGAGTATGACATTTTTTATTTAGAAAAACTCATCGAGCATCAAATG
OAJ31	CGCAAGCGGTCACTCTATCAG
OAJ32	CATACAGCGACGAGGTGGAAC
OAJ74	GCCGATCGCAAGGTGAATCAG
OAJ54	CGCCTGTCAATAAACCGACAC

Table S3 (excel file): Correlation between ATP levels and RNA stability. ATP levels and the half-lives of 12 selected RNAs were performed in the presence of no, low (0.7 mM), and high (2–3 mM) concentrations of DNP in M9-succinate for strains MG1655, MG1655Δ*pcnB*, RhlB^{+}, RhlB^{-}, MG1655Δ*pcnB*/p, and MG1655Δ*pcnB*/pPAP I. The half-life of each RNA was normalized by its value in MG1655 grown without DNP.

Table S4: Effect of read cutoff values on RNA half-life determination. RNA half-lives ($t_{1/2}$) were obtained from the linear regression coefficient, k, of $\log(\mathrm{RNA}) = \log(a_0) - k * t$, with a_0 the RNA concentration à T0, using the relationship $t_{1/2}$ = 1n2/k. RNA half-lives were only considered reliable if the associated R^2 was >70%. Half-lives were compared using t-tests, and differences were considered statistically significant if the adjusted *P* value was <0.05.

	10 read cutoff	100 read cutoff
RNA with reliable half-life in MG1655 and MG1655Δ*pcnB*	2627	1973
Stabilized RNA in MG1655Δ*pcnB* versus MG1655	1403	1142
Destabilized RNA in MG1655Δ*pcnB* versus MG1655	4	2
RNA with reliable half-life in MG1655 treated with 0.7 mM and 2 mM DNP	2189	1635
Stabilized RNA at 2 mM DNP versus 0.7 mM DNP	1769	1422
Destabilized RNA at 2 mM DNP versus 0.7 mM DNP	15	12

Supplementary Figure S1. Effects of delay and residual RNA concentration on the determination of RNA half-life. RNA half-lives determined using a simple linear model as in this study were compared with those obtained when considering a delay α before the onset of the exponential decay with a residual RNA concentration b and without (b = 0) at the end of the decay process, according to the following equations (1, 2):

$$\mathrm{RNA}(t) = a_0, \text{ if } 0 < t < \alpha,$$

$$\mathrm{RNA}(t) = b + (a_0 - b)e^{-k*(t-\alpha)}, \text{ if } t \geq \alpha.$$

The parameters a_0, b, k, and α were estimated using the nls function of the R programming language after log transformation of RNA levels. Half-lives were considered as accurate enough when the coefficient of variation of RMSE divided by the mean of the observed values (RMSE being the sum of the square roots of the residuals of the model divided by the number of values) was below 0.3.

In Figure S1, RNA half-lives without delay were plotted against RNA half-lives with a delay estimated with or without b in the two strains, MG1655 and MG1655Δ*pcnB*. In Figure S1A, only a delay before the onset of the exponential decay (b=0) was considered. The insets are histograms of the delay values. In Figure S1B, a delay before the onset of the exponential decay plus a residual RNA concentration b at the end of the decay process were considered. The insets are histograms of the b/a_0 values. The half-lives estimated with delay were compared between the two strains, MG1655 and MG1655Δ*pcnB*, in box plots, where data points are shown in black, outliers in gray, and median values in red. Wilcoxon–Mann–Whitney test, ***P < 0.001. The linear regression line is shown in blue.

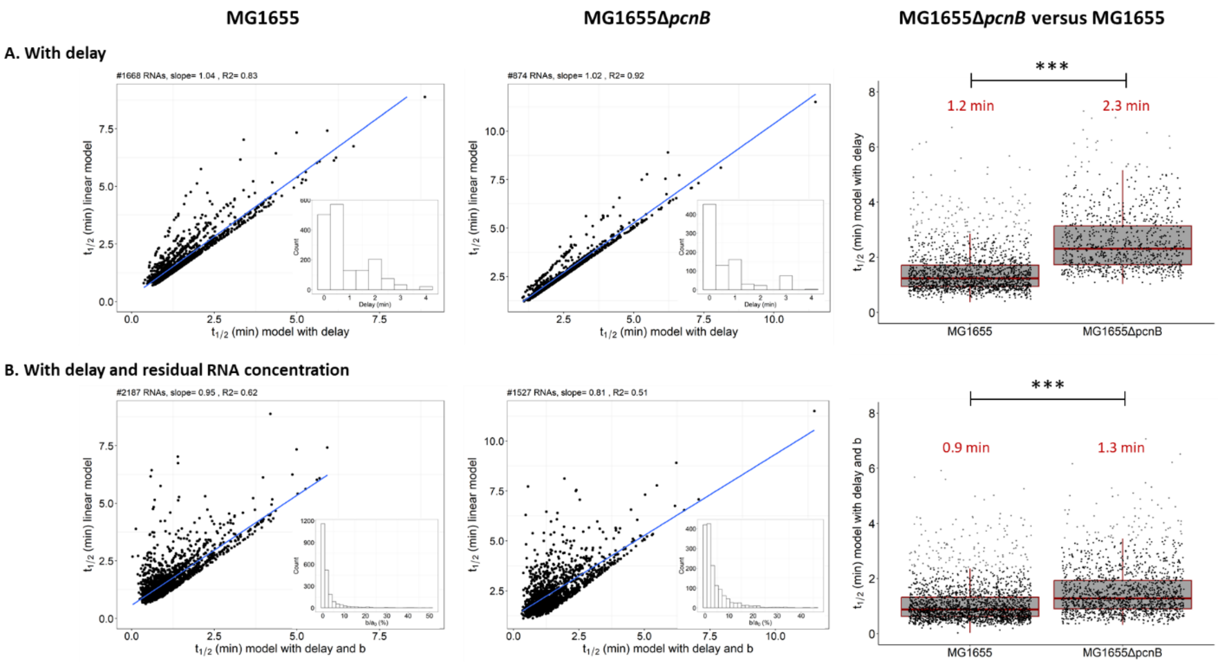

MG1655
MG1655Δ*pcnB*
MG1655Δ*pcnB* versus MG1655
A. With delay
#1668 RNAs, slope= 1.04 , R2= 0.83
#874 RNAs, slope= 1.02 , R2= 0.92
t1/2 (min) linear model
t1/2 (min) model with delay
Delay (min)
Count
***
1.2 min
2.3 min
t1/2 (min) model with delay
MG1655
MG1655ΔpcnB
B. With delay and residual RNA concentration
#2187 RNAs, slope= 0.95 , R2= 0.62
#1527 RNAs, slope= 0.81 , R2= 0.51
t1/2 (min) linear model
t1/2 (min) model with delay and b
***
0.9 min
1.3 min
t1/2 (min) model with delay and b
MG1655
MG1655ΔpcnB

Supplementary Figure S2. Semilogarithmic plots illustrating the degradation profiles of stabilized and destabilized RNAs between MG1655 and MG1655Δ*pcnB* in M9-glucose. RNA-seq was used to analyze samples of total cellular RNA extracted at various times after inhibiting transcription with rifampicin. The time points of three independent degradation kinetics were interleaved and the linear regression coefficient, *k*, of ln(RNA) versus time was calculated over 12 points. RNA half-lives ($t_{1/2}$) were obtained from *k* using the relationship $t_{1/2} = \ln 2/k$. MG1655 is in blue and MG1655Δ*pcnB* in orange. The three independent degradation kinetics are represented by squares, triangles, and circles. A. Examples of stabilized RNA between MG1655 and MG155Δ*pcnB*. B. The four destabilized RNAs between MG1655 and MG1655Δ*pcnB*.

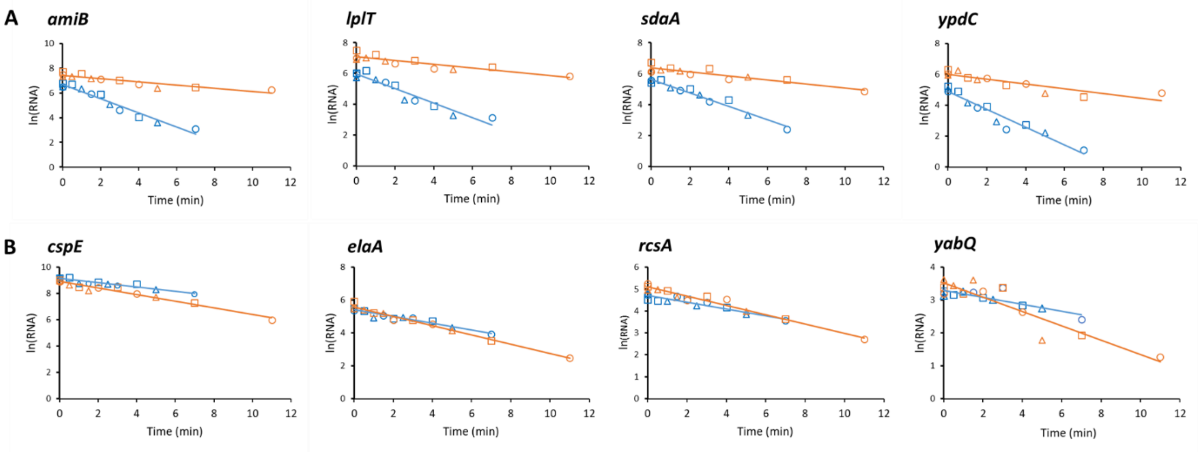

Supplementary Figure S3. Stabilized RNA molecules in the *pcnB* mutant. Using RNA-seq data for RNAs stabilized in MG1655Δ*pcnB versus* MG1655, we produced 3D plots of surfaces of raw counts along the gene as a function of time after the addition of rifampicin in the two strains. Representative examples of RNA stabilization are shown. A. Stabilization of a full-length RNA molecule (*rpmH*). B. Stabilization of a 5′ end RNA fragment (*yraN*). C. Stabilization of a fragment within the RNA (*acrF*). D. Stabilization of a 3′ end RNA fragment (*zapB*).

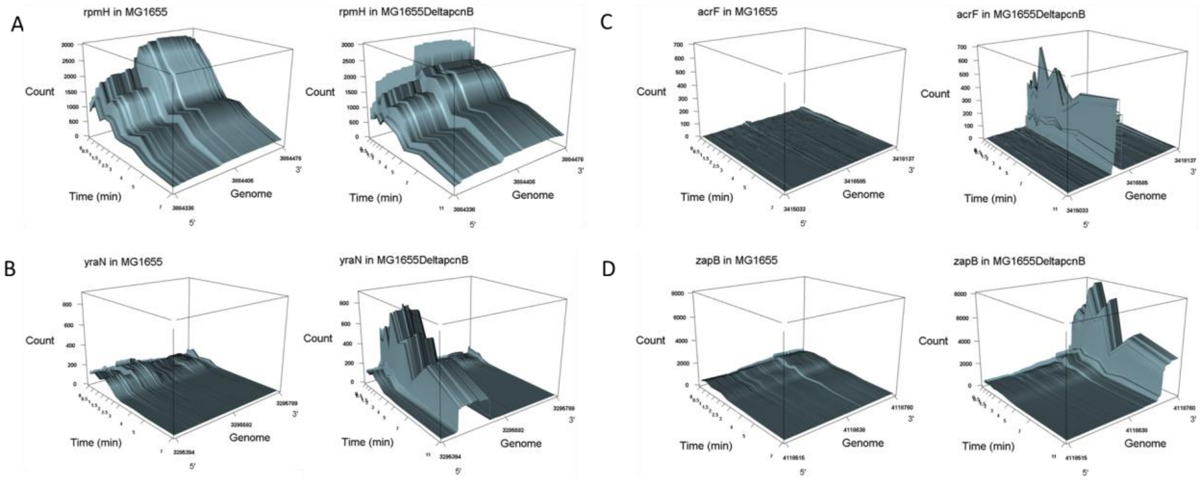

Supplementary Figure S4. *zapB* northern blot analysis. A. Northern blots after rifampicin addition (at times T0, 1.5, 3, and 7 min) of total RNA (10 µg) of MG1655Δ*pcnB* and MG1655, probed for *zapB*. B. Northern blots after rifampicin addition (at times T0, 1, 4, and 7 min) of total RNA (2.5 µg) of MG1655 cells treated with 0.7 and 2 mM DNP, probed for *zapB*. For each sample, total RNA was denatured for 10 min at 37°C and 550 rpm with MultiTherm (model H5000-HC-E from Benchmark, 550 rpm) in RNA loading buffer (95% [v/v] formamide, 0.1% [w/v] xylene cyanole, 0.1% [w/v] bromophenol blue, 10 mM EDTA, SDS 0.025%), separated on a 2% agarose gel (TBE) at 100 V for 45 min. Gel was ethidium bromide stained for loading visualization based on rRNA signal, and then passively transferred to Hybond-XL membranes (Amersham) (o/n, TBE 1×). After UV crosslinking, the membranes were hybridized overnight in 10 mL Roti®-Hybri-Quick (Roth) at 68°C with 50 ng/mL biotinylated synthetic RNA *zapB* probe (see below for synthesis). The hybridization was followed by 15 min washes in SSC 5× and 1× and 8 min in 0.1×/0.1% SDS solutions at 68°C. Hybridization signals were quantified with Chemiluminescent Nucleic Acid Detection Module (Thermo Fisher) with Chemidoc MP (Biorad). To generate biotinylated RNA probe, the reverse strand of the *zapB* CDS was PCR amplified from MG1655 genomic DNA using a primer pair including T7 promoter sequence (GAAATTAATACGACTCACTATAGGGTCAGACCTCTTCCATGCGACC ATGACAATGTCATTAGAAGTGTTTGAG). Amplicon was gel purified (QIAquick Gel Extraction Kit, Qiagen) and *in vitro* transcribed (High-Fi RNA polymerase, NEB) in a mix containing 3.5 mM Biotin-16-UTP (Roche) according to manufacturer's recommendations. DNA matrix was removed with DNase treatment (NEB). The RNA probe was purified by phenol–chloroform precipitation and quantified by NanoDrop. The methods employed are adapted from (3). M: molecular marker (100 bp NEB).

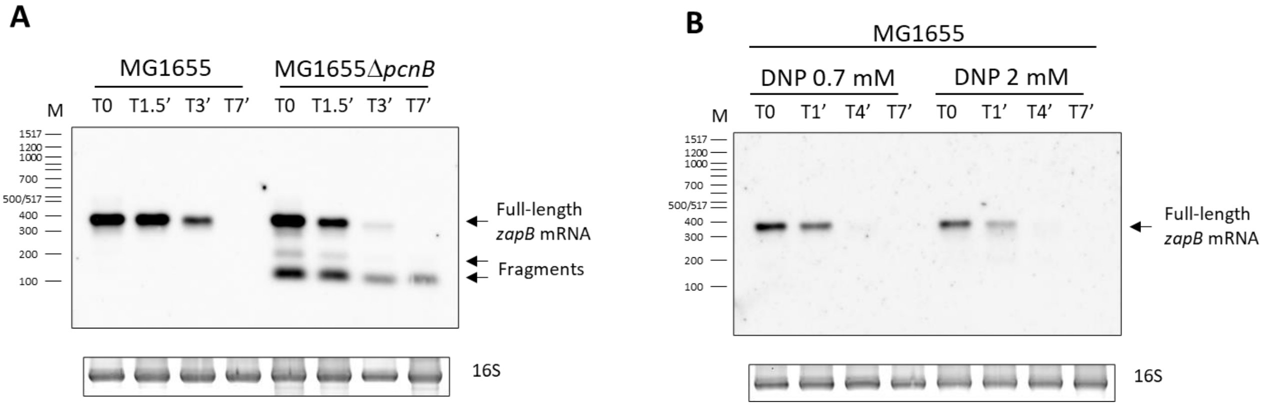
A
MG1655
MG1655Δ*pcnB*
M
T0
T1.5'
T3'
T7'
1517
1200
1000
700
500/517
400
300
200
100
Full-length
zapB mRNA
Fragments
16S
B
MG1655
DNP 0.7 mM
DNP 2 mM
T1'
T4'

Supplementary Figure S5. Plots of quantification cycle (Cq) versus time illustrating RNA degradation profiles. Quantitative RT-PCR was used to quantify RNA concentration at various times after inhibiting transcription with rifampicin. In each condition, two independent kinetics of six time points are shown. RNA half-lives ($t_{1/2}$) were obtained from the linear regression coefficient, k', using the relationship $t_{1/2} = 1/k'$.

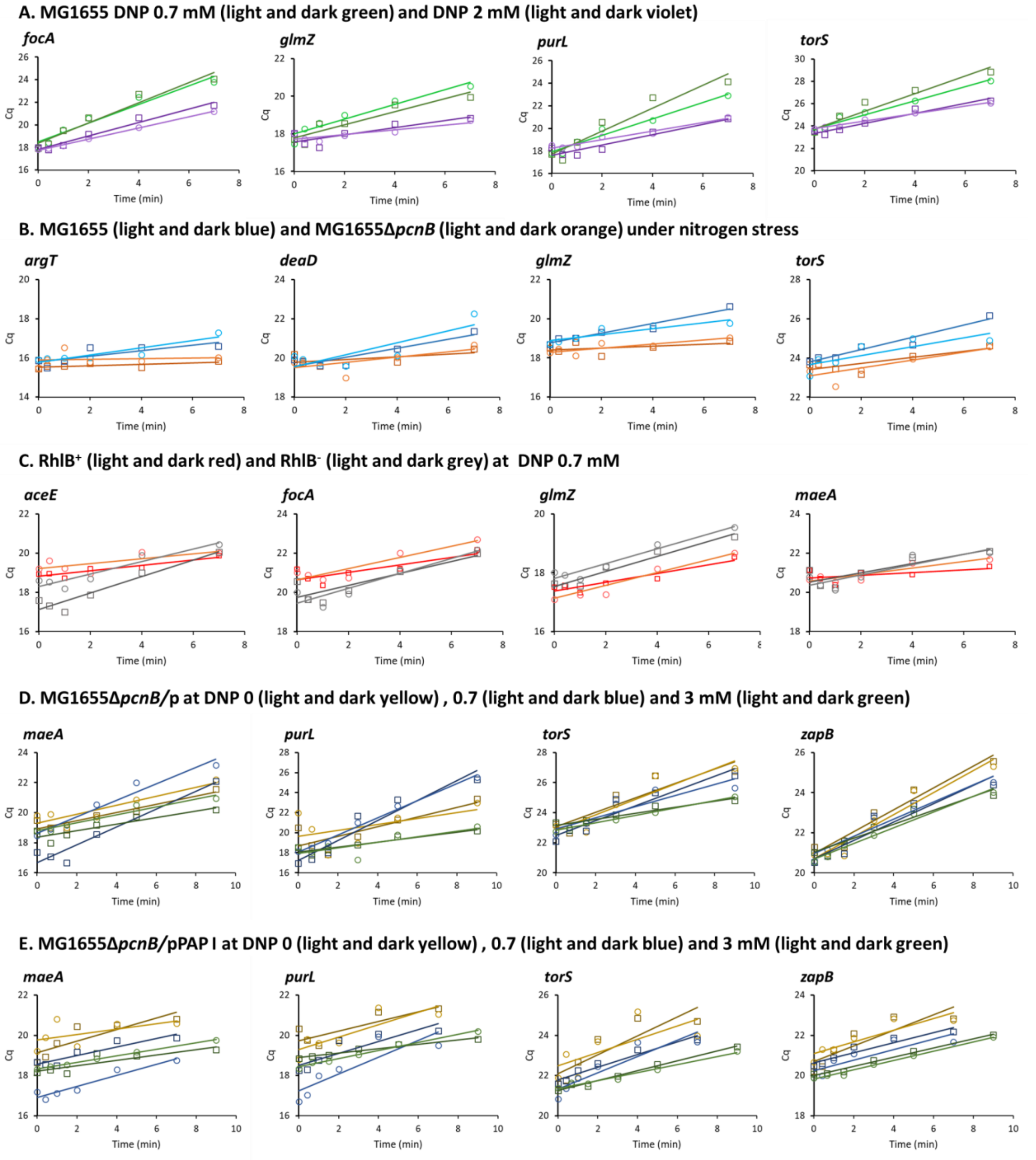

Supplementary Figure S6. Stabilized RNA molecules in MG1655 at low ATP levels. Using RNA-seq data for RNAs stabilized in MG1655 cells treated with 2 mM DNP compared to 0.7 mM, we produced 3D plots of surfaces of raw counts along the gene as a function of time after the addition of rifampicin. Two types of RNA stabilization are shown. A. Stabilization of a full-length RNA molecule (*zapB*). B. Stabilization of a 5′ end RNA fragment (*focA*).

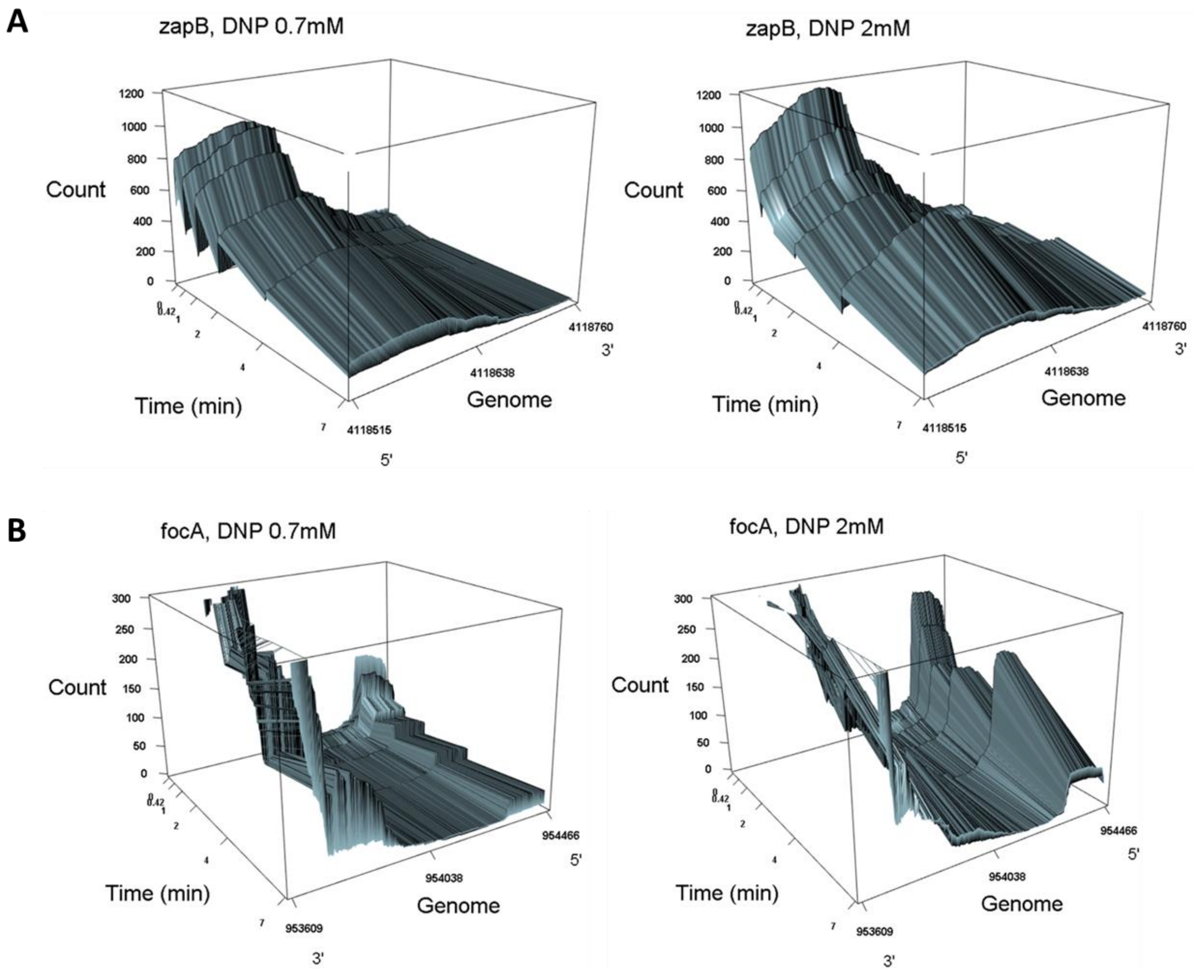

Supplementary Figure S7. Genome-wide RNA stabilization induced at low ATP levels. A**.** Boxplots of RNA half-lives in *E. coli* MG1655 cells treated with 0.7 and 2 mM DNP, for 2,189 RNAs with reliably determined half-lives in both conditions. Data points on box plots are shown with black filled circles and outliers with black open circles. Wilcoxon–Mann–Whitney test, ****P* < 0.001. B. Volcano plot of the log2 fold change (logFC) of RNA half-lives between *E. coli* MG1655 cells treated with 2 mM DNP versus 0.7 mM DNP. The values above the horizontal black line correspond to significant differences in fold change ($P \leq 0.05$). The 1,769 RNAs stabilized with 2 mM DNP at low ATP levels are shown in red. The 15 RNAs destabilized are shown in green. The 405 RNAs with non-significant differences in stability are shown in gray.

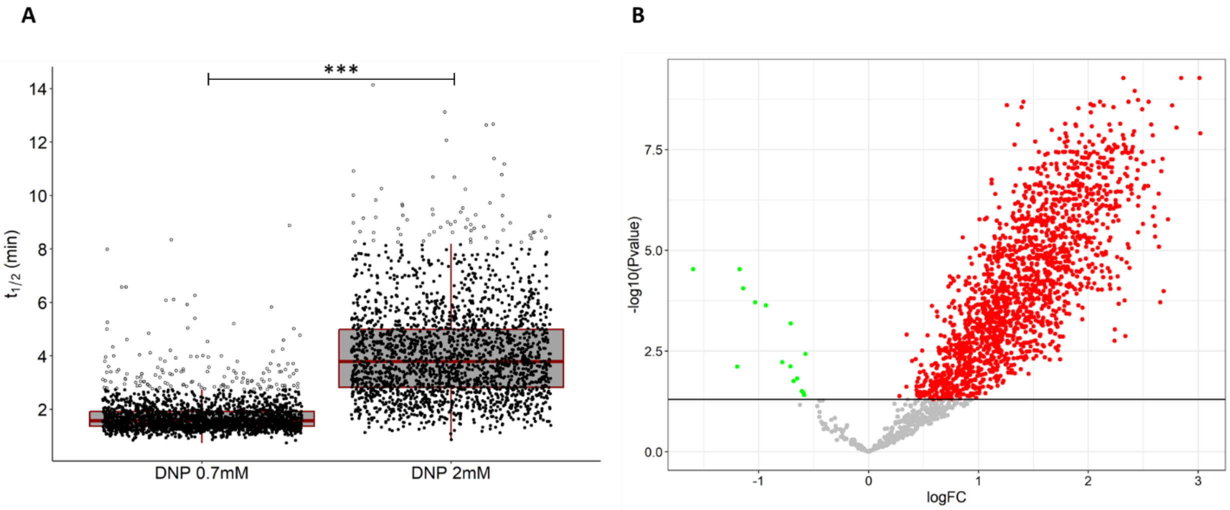

References

1. Chen H, Shiroguchi K, Ge H, Xie XS. 2015. Genome-wide study of mRNA degradation and transcript elongation in Escherichia coli. Mol Syst Biol 11:781.
2. Moffitt JR, Pandey S, Boettiger AN, Wang S, Zhuang X. 2016. Spatial organization shapes the turnover of a bacterial transcriptome. Elife 5.
3. McKenney KM, Connacher RP, Dunshee EB, Goldstrohm AC. 2024. Chemi-Northern: a versatile chemiluminescent northern blot method for analysis and quantitation of RNA molecules. RNA 30:448-462.
